# Infant functional networks are modulated by state of consciousness and circadian rhythm

**DOI:** 10.1162/netn_a_00194

**Published:** 2021-06-21

**Authors:** Rachel J. Smith, Ehsan Alipourjeddi, Cristal Garner, Amy L. Maser, Daniel W. Shrey, Beth A. Lopour

**Affiliations:** Department of Biomedical Engineering, University of California, Irvine, CA, USA; Department of Biomedical Engineering, University of California, Irvine, CA, USA; Division of Neurology, Children’s Hospital of Orange County, Orange, CA, USA; Department of Psychology, Children’s Hospital of Orange County, Orange, CA, USA; Division of Neurology, Children’s Hospital of Orange County, Orange, CA, USA; Department of Pediatrics, University of California, Irvine, Irvine, CA, USA; Department of Biomedical Engineering, University of California, Irvine, CA, USA

**Keywords:** Functional connectivity, Graph theory, Cross-correlation, Electroencephalography, Resting-state networks, Pediatrics

## Abstract

Functional connectivity networks are valuable tools for studying development, cognition, and disease in the infant brain. In adults, such networks are modulated by the state of consciousness and the circadian rhythm; however, it is unknown if infant brain networks exhibit similar variation, given the unique temporal properties of infant sleep and circadian patterning. To address this, we analyzed functional connectivity networks calculated from long-term EEG recordings (average duration 20.8 hr) from 19 healthy infants. Networks were subject specific, as intersubject correlations between weighted adjacency matrices were low. However, within individual subjects, both sleep and wake networks were stable over time, with stronger functional connectivity during sleep than wakefulness. Principal component analysis revealed the presence of two dominant networks; visual sleep scoring confirmed that these corresponded to sleep and wakefulness. Lastly, we found that network strength, degree, clustering coefficient, and path length significantly varied with time of day, when measured in either wakefulness or sleep at the group level. Together, these results suggest that modulation of healthy functional networks occurs over ∼24 hr and is robust and repeatable. Accounting for such temporal periodicities may improve the physiological interpretation and use of functional connectivity analysis to investigate brain function in health and disease.

## Author Summary

In adults, functional connectivity networks are modulated by the state of consciousness and the circadian rhythm. We investigated whether infant brain networks exhibited similar variations, given the unique temporal properties of infant sleep and circadian patterning. Functional connectivity networks were calculated from ∼24-hr EEG recordings from 19 healthy infants. We found that networks were subject specific and exhibited stronger connections during sleep compared to wakefulness. Within subjects, both sleep and wake networks reoccurred over time. However, network strength, degree, clustering coefficient, and path length significantly varied with time of day, when measured in either wakefulness or sleep at the group level. Together, these results suggest that modulation of healthy infant functional networks occurs over 24-hr periods and is robust and repeatable.

## INTRODUCTION

Measurements of functional connectivity in the infant brain can provide valuable insight into neural development, cognition, and disease. Much of our knowledge of infant brain networks comes from functional magnetic resonance imaging (fMRI) studies (Gao et al., 2017), but EEG is increasingly being used to probe such questions, as it has the advantage of sampling functional networks with high temporal resolution for long periods of time. For example, EEG-based functional networks reflect the network segregation that occurs during prenatal development, with newborns exhibiting network hubs in the frontal and parieto-occipital lobes (Omidvarnia et al., 2014; Tóth et al., 2017). These networks evolve slowly throughout childhood, with unique frequency-specific connectivity for infants, toddlers, children, and adolescents (Chu et al., 2014). EEG-based functional networks are also associated with cognitive functions, such as sustained infant attention (Xie, Mallin, & Richards, 2019), and network oscillations during sleep are correlated with motor, language, and social development in toddlers (Page, Lustenberger, & Fröhlich, 2018). As a marker of disease, functional connectivity analysis has been used to characterize infantile epilepsy (Shrey et al., 2018) and predict the onset of autism spectrum disorder (Righi et al., 2014).

To accurately assess these functional networks, the subject’s state of consciousness must be considered. In adults, functional networks measured during wakefulness exhibited higher density and lower clustering coefficient than those measured during sleep (Chu et al., 2012), such that networks during sleep exhibit small-world properties (Ferri et al., 2008). In contrast, infant networks exhibit greater strength during sleep compared to wakefulness, for both healthy subjects and those with epilepsy (Smith et al., 2020). Moreover, the network characteristics are a function of the specific sleep stage. In newborns, studies found that strong, short-range functional connections are concentrated in the occipital lobe during active sleep, with more broadly distributed long-distance connections predominating during quiet sleep (Tokariev et al., 2019; Tokariev, Vanhatalo, & Palva, 2016). Despite the widespread accessibility of EEG and its broad use in the young, no prior studies of infant sleep networks have included analysis of overnight EEG recordings. Most have relied on short clips, ∼3–20 min in duration, recorded during daytime sleep. Only one study (Smith et al., 2020) included a direct comparison of networks during sleep and wakefulness. Filling this knowledge gap is critical for understanding the role that functional networks play in health and disease.

Beyond the characteristics associated with each discrete state of consciousness, features of the EEG exhibit periodicities over a range of timescales. Ultradian periodicities in EEG signal features, such as frequency band power, have been well studied in adults (Aeschbach et al., 1999; Kaiser, 2008). It is known that EEG-based functional networks are stable when measured over several minutes in sleep or wakefulness, despite the rapid visual variability of the time series (Chapeton, Inati, & Zaghloul, 2017; Kramer et al., 2011). However, functional networks based on intracranial EEG exhibit strong circadian modulation of graph theoretical measures, such as the clustering coefficient and path length (Geier, Lehnertz, & Bialonski, 2015; Kuhnert, Elger, & Lehnertz, 2010). This is emerging as a promising marker to aid in seizure prediction, as oscillations in functional network properties over hours and days have been shown to correlate to seizure onset in patients with epilepsy (Anastasiadou et al., 2016; Baud et al., 2018; Karoly et al., 2017; Kuhnert et al., 2010; Mitsis et al., 2020). However, ultradian and circadian periodicities of infant functional networks have yet to be studied. Given that infant sleep cycles are quite distinct from adult sleep patterns, in both electrographic appearance and circadian patterning, the networks may exhibit modulation over timescales that are less than 24 hr, or not at all. These unknown temporal periodicities have the potential to further confound the study of infant brain networks.

We hypothesize that both the state of consciousness and the time of day will significantly impact spontaneous functional networks in infants. Therefore, our overall goal is to use ∼24-hr EEG recordings from healthy subjects to disentangle these effects so they can be accounted for in future studies. To do this, we take advantage of the noncircadian infant sleep cycle, which allows us to record periods of wakefulness and sleep during both the day and the night. We first describe the functional networks associated with sleep and wake states, assess the intersubject variability in those state-specific networks, and identify graph theoretical measures that separate the two states. Then we show that measurement of each sleep or wake network is stable and repeatable within an individual subject. Lastly, we show circadian variation in functional connectivity strength and graph theoretical measures when assessed at the group level. This work increases our understanding of the infant brain’s physiological fluctuations in functional connectivity, which has the potential to act as a baseline for investigations of development, cognition, and disease.

## METHODS

### Subject Recruitment and EEG Recording

This prospective study was approved by the Institutional Review Board of the Children’s Hospital of Orange County. Subjects were recruited and consented from June 2017 to February 2019 and underwent overnight long-term video EEG recording to rule out a form of pediatric epilepsy called infantile spasms. If the infant was not diagnosed with infantile spasms, they were classified as a control subject. Clinical data were collected at the time of enrollment. Subjects were deemed “healthy” controls if they (a) exhibited a normal EEG recording, (b) did not receive a diagnosis of epilepsy, (c) had no known neurological conditions, and (d) were developmentally normal for age (as assessed with the Vineland Adaptive Behavior Scales, 3rd Edition, Sparrow, Cicchetti, & Saulnier 2016). Nineteen channels of EEG data were sampled at 200 Hz with impedances below 5 kΩ.

A certified sleep technologist at the Children’s Hospital of Orange County manually delineated time periods of wakefulness, rapid eye movement (REM) sleep, and non-REM sleep stages (N1, N2, N3) in all EEG recordings in accordance with the American Academy of Sleep Medicine guidelines. For our analysis, time periods of sleep and wakefulness were separated based on these markings. For comparison with automatic sleep staging (see section Recurrence of Network States and Correspondence to Manual Sleep Staging), we combined N1, N2, N3, and REM sleep stages into one “sleep” category.

### EEG Preprocessing

EEG data were re-referenced offline to the common average. To be effective, this referencing scheme relies on substantial coverage of the head by electrodes; in adults, this may imply the use of high-density EEG. However, infant heads are significantly smaller than those of adults, thus providing more extensive coverage than is usually possible with 19 electrodes. In addition, the connectivity measure used here (cross-correlation measured at nonzero time lags; see section Functional Connectivity) was found to be minimally affected by the choice of reference, with common average, bipolar, and common referencing schemes providing similar results (Anastasiadou et al., 2019).

Artifactual time periods were identified with an automatic extreme value artifact detector, similar to previously published methods (Durka et al., 2003; Moretti et al., 2003). Specifically, to identify artifacts we broadband bandpass filtered the data (1.5–40 Hz, Butterworth filter, chosen to match the settings of clinical EEG viewing/analysis), subtracted the mean from each channel, and calculated the standard deviation of each zero-mean time series. Artifacts were defined as time points in which the absolute value of the voltage exceeded a threshold of 7.5 standard deviations above the mean value in any single channel. We chose this threshold because it resulted in the best correspondence between automatically detected and visually identified artifacts in a previous dataset (Smith et al., 2017). To ensure that the entire artifact was marked, a buffer of 0.9 s was added to both sides of each contiguous set of time points containing extreme amplitude values. Data recorded during EEG impedance checks were also marked as artifact. For the connectivity analysis, a broadband bandpass filter was applied to the raw, re-referenced data (0.5–55 Hz, Butterworth filter). One-second epochs that contained artifactual data were removed from all channels after filtering.

### Functional Connectivity

We calculated functional connectivity networks via cross-correlation using the method developed by Kramer et al. (2009) and Chu et al. (2012) and previously applied to infant EEG data (Shrey et al., 2018). We chose cross-correlation because it is a simple bivariate measure that is highly sensitive to linear changes in EEG activity (David, Cosmelli, & Friston, 2004; Jalili, Barzegaran, & Knyazeva, 2014) and has been shown to be comparable to other measures of synchronization (Jalili et al., 2014; Quian Quiroga et al., 2002). Although cross-correlation is generally insensitive to nonlinear interactions in the EEG, we opted for this rapid and straightforward linear measure of synchronization because no nonlinear metric has been shown to reliably measure actual changes in coupling strength while discounting spurious increases in synchronization due to changes in other signal properties (David et al., 2004; Pereda et al., 2001).

The functional connectivity calculation was performed as described in Shrey et al. (2018); we briefly summarize it here. Data were divided into 1-s epochs, and the EEG signals in each epoch (one from each channel) were normalized to have zero-mean and unit variance. For each epoch, we calculated the cross-correlation between every pair of channels and identified the maximum of the absolute value of the cross-correlation. Epochs in which the maximal cross-correlation value occurred at zero time lag were excluded, as they were likely a result of volume conduction (Chu et al., 2012). A partial correlation with the common average reference time series was performed to test whether the reference induced the correlation measured between the channels (Shrey et al., 2018). If there was a large difference between the partial correlation and the correlation value between the channels, the measured correlation was presumed to have resulted from referencing artifact and the epoch was removed from further analysis (Supporting Information Table S1). Z-values were calculated for the nonartifactual epochs by dividing the Fisher-transformed correlation coefficient value by the estimated standard deviation, taking the autocorrelation of each channel epoch into account (Chu et al., 2012; Kramer et al., 2009). This method nullifies the autocorrelation component of the time series that would produce spurious correlations. The z-values were compared to a baseline distribution created via permutation resampling. Permutation resampling was performed by selecting two random 1-s epochs of data from the time series that were separated by at least 1 s, calculating the max cross-correlation between the channels, and iterating this procedure 500 times (Nichols & Holmes, 2003). The standardized correlation values from all iterations were sorted and the threshold of significance was defined as the value corresponding to the 95th percentile of the distribution for each electrode pair. For each epoch, correlation values between channel pairs that exceeded this threshold value were deemed to be significant, and these connections were assigned a value of one; connections that did not exceed this threshold were assigned a value of zero. Thus for *p* EEG channels, the output of the connectivity calculation for each epoch was a binary matrix of dimension *p* × *p*.

Across epochs, connectivity data were stored in a three-dimensional array ***Q***, where the binary value at position ***Q***(*i*, *j*, *k*) represented the connection between electrode *i* and electrode *j* in epoch *k*. Then the overall connection strength between two channels was calculated as the fraction of time series epochs in which there was a significant connection between them. For wakefulness, this calculation used the *N*_*w*_ binary connectivity matrices that coincided with times of wakefulness, based on manual sleep staging. For sleep, the *N*_*s*_ epochs marked as sleep (N1, N2, N3, and REM) were used. Because *N*_*w*_ and *N*_*s*_ were unequal for each subject, we performed a bootstrapping procedure. For each iteration, we randomly sampled 11,000 epochs (*N*_*samp*_) with replacement from each sleep/wake state; we chose 11,000 epochs because this was the shortest duration of a single sleep/wake state over all patients. We used those values to calculate functional connectivity networks associated with wakefulness, $Q_{w}^{m}$, and sleep, $Q_{s}^{m}$. This constituted one iteration, denoted by the superscript *m*. Specifically, $Q_{w}^{m}$(*i*, *j*) = (1/*N*_*samp*_) ∑_*k*∈*wake*_ *Q*(*i*, *j*, *k*) and $Q_{s}^{m}$(*i*, *j*) = (1/*N*_*samp*_) ∑_*k*∈*sleep*_ *Q*(*i*, *j*, *k*). We performed this calculation for 1,000 iterations and then averaged to obtain the wakefulness network ***Q***_*w*_ and the sleep network ***Q***_*s*_ for each subject, where ***Q***_*w*_(*i*, *j*) = (1/1,000) $\sum_{m=1}^{1,000}$ $Q_{w}^{m}$ and ***Q***_*s*_(*i*, *j*) = (1/1,000) $\sum_{n=1}^{1,000}$ $Q_{s}^{m}$. We evaluated network strength by applying a proportional threshold to the connectivity values (Garrison et al., 2015). Specifically, we calculated the average of the strongest 10% of connections in the wakefulness and sleep networks. The Benjamini–Hochberg procedure was used to correct for multiple comparisons where applicable (Benjamini & Hochberg, 1995).

### Graph Theoretical Measures

Studies of functional connectivity are often augmented by complex network analysis because it provides easily computable measures with biophysiological significance (Rubinov & Sporns, 2010). We computed three weighted graph theoretical measures that summarize the functional connectivity networks computed in this study. First, we calculated the *degree* for each node by summing the weights of the connections incident to that node (Bullmore & Sporns, 2009; Rubinov & Sporns, 2010). The degree is related to our measurement of network strength, which is an important marker for distinguishing sleep and wakefulness (Smith et al., 2020) and can also be an indicator of pathological networks (Shrey et al., 2018). However, the weighted calculation of degree has an advantage over strength, as it does not require a threshold to binarize the network. Second, the *clustering coefficient* is defined as the fraction of a node’s neighbors that are also neighbors of each other and is thought to quantify the level of functional segregation in the brain network (Rubinov & Sporns, 2010; Watts & Strogatz, 1998). In the weighted network case, the clustering coefficient is derived from the “intensity” and “coherence” of a subgraph using measures of its geometric and arithmetic mean (Onnela et al., 2005). Before calculating the clustering coefficient, we normalized the adjacency matrix by dividing each element by the maximum connection value in the network (Antoniou & Tsompa, 2008; Onnela et al., 2005). Third, we computed the *shortest path length*, which reports the minimum sum of the edge “lengths” for a path from one node to another (Antoniou & Tsompa, 2008; Rubinov & Sporns, 2010; Stam & Reijneveld, 2007). In our case, because we assume that information flow will increase with higher connection values, we defined the edge lengths as the inverse of the edge weights. Thus, the minimum sum of these inverse edge weights maximizes the connectivity strength between each pair of electrodes (Rubinov & Sporns, 2010). The shortest path length is one of the most common metrics to assess functional integration (Rubinov & Sporns, 2010). Similar to the clustering coefficient calculation, we normalized the adjacency matrix before computing the inverse and determining the shortest paths between nodes in the network (Antoniou & Tsompa, 2008; Onnela et al., 2005).

We chose these measures because the clustering coefficient and characteristic path length have previously been used to characterize the newborn and infant brain (Omidvarnia et al., 2014; Tymofiyeva et al., 2012). Clustering coefficients decrease in lower frequency bands and increase in higher frequency bands during development (Chu et al., 2014; Omidvarnia et al., 2014; Tymofiyeva et al., 2012). Path length increases and clustering coefficients decrease with sustained attention in infants (Xie et al., 2019), and the ratio between the clustering coefficient and path length indicates that the infant brain exhibits small-world properties (Gao et al., 2011; Tymofiyeva et al., 2012). Moreover, changes in brain state affect graph theoretical measures in adults, with higher clustering coefficients (Chu et al., 2012) and greater small-worldness (Ferri et al., 2008) during sleep. Importantly, such global graph characteristics can be robustly measured in infant EEG, as indicated by a test-retest reliability study (van der Velde, Haartsen, & Kemner 2019), making them suitable for our analysis.

### Time-Varying Functional Connectivity Measurement

To analyze time-varying changes in the functional connections, we averaged the binary *p* × *p* matrices across windows of successive epochs. Let ***Q***_*n*_ represent the *p* × *p* matrix averaged over a window of *n* 1-s epochs. The value of ***Q***_*n*_(*i*, *j*) indicates the proportion of epochs in which the connection between channel *i* and channel *j* was significant, analogous to the values of ***Q***_*w*_ and ***Q***_*s*_ for wakefulness and sleep, respectively. For our analysis, we calculated ***Q***_**300**_, the averaged connectivity matrix in a window of 300 s, and this window was shifted in 30-s increments (90% overlap). We chose a window size of 300 s because networks were shown to be stable over this amount of time in two separate studies (Chu et al., 2012; Shrey et al., 2018).

### Recurrence of Network States and Correspondence to Manual Sleep Staging

We hypothesized that different brain states (e.g., sleep or wakefulness) would be associated with different functional networks and that the functional network associated with a single state would be relatively consistent over time. Therefore, we used principal component analysis (PCA) to identify the set of functional networks that accounted for a majority of the variance over time, assuming that state transitions would be the greatest source of this variance. This also let us assess how many network states occurred in the data and whether the states recurred over time.

To perform PCA on the functional connectivity networks over time, we first calculated ***Q***_**300**_ in 300-s windows with 90% overlap, as described in section Time-Varying Functional Connectivity Measurement. We then placed the values for all unique connections in ***Q***_**300**_ (171 channel pairs in total, excluding self-connections) into a column vector ***c*** and normalized it to have zero-mean and unit variance. We concatenated *p* successive vectors, where *p* denotes the number of windows that were available in the dataset, into matrix ***C*** and subtracted the mean from each row to ensure that the distribution of connections for each channel pair was zero-mean. We performed PCA on these normalized functional connectivity time series ***C*** to ascertain the latent networks that explained the most variance in the data. We then calculated the time course of the first principal component, which represents the relative weight assigned to that component as a function of time.

Once the network states were identified, we determined their physiological significance by comparing them to visual sleep staging of the EEG. We hypothesized that there would be transitions between two different states, likely representing wakefulness and sleep, so we fit a two-component Gaussian mixture model (GMM) to the principal component time series. We then calculated normalized probability distribution functions (PDFs) for the two GMM distributions. The threshold to separate the states was defined as the intersection of the two PDFs. To avoid finding intersections at the extreme tails of the distributions, we calculated the PDF ratio and identified the index where this ratio was closest to 1:

$$\text{thresholdIndex}=\min\left(\left|\frac{{PDF}_{1}}{{PDF}_{2}}-1\right|\right).$$

The principal component value associated with this index was the threshold that best distinguished the two states. We used this value to separate the networks from all time points into two states, and we compared these results to visual sleep scoring done by a certified EEG sleep technician.

### Calculation of Network Stability

We assessed network stability of the functional connectivity measurement by performing 2-D correlations between independent average connectivity networks during wakefulness or sleep. We first concatenated all epochs during sleep (*N*_*s*_) or wakefulness (*N*_*w*_) and then calculated *N*_*s*_/*n* or *N*_*w*_/*n* sequential, nonoverlapping measurements of ***Q***_*n*_ where *n* is the size of the window. Then a 2-D correlation was calculated between each successive measurement of ***Q***_*n*_ and this was repeated for window sizes *n* ranging from 10 s to 200 s. The mean of the correlation values was recorded for each window size for each subject. The mean and 95% confidence interval of the average correlation coefficient values for all subjects were then plotted as a function of *n*.

### Calculation of Circadian Changes in Functional Networks

Lastly, we investigated whether there were circadian modulations in the functional connectivity networks. For this analysis, sleep and wakefulness were analyzed separately based on manual EEG sleep scoring. For both wakefulness and sleep, we calculated four metrics (the mean network strength and the three graph theoretical measures) as a function of time using ***Q***_**300**_ (see section Time-Varying Functional Connectivity Measurement). Next, the values of each of these four metrics were associated with the 24-hr (circadian) clock time corresponding to the beginning of their ***Q***_**300**_ epoch. We did this for each subject in the dataset, thereby obtaining a distribution of values across subjects for each of the 1,440 circadian time points (every minute on a 24-hr clock). We then calculated the mean across all subjects for each 1-min block of time; if there were less than five data points in the 1-min block, the data were considered insufficient and that time point was discarded. To assess statistical significance, we aggregated all data points from 11 AM to 1 PM as the “daytime” distribution and we aggregated all data points from 11 PM to 1 AM as the “nighttime” distribution. Similar to the sampling methods used in section Functional Connectivity, we randomly selected observations with replacement from the daytime and nighttime periods for 1,000 iterations. With each iteration, we calculated the difference between the means of the distributions (daytime compared to nighttime), and we tested whether the confidence interval of the resulting difference distribution included 0.

## RESULTS

### Subject Demographics

In total, 19 healthy subjects (15 female, 4 males) were recruited for the study between June 2017 and February 2019, and all were included in our analysis. The mean age of the subjects was 6.3 months (+/− 3.1 months, standard deviation). The recordings lasted 20.8 hr on average (+/− 7.8 hr, standard deviation). Recording durations are listed for every subject in Supporting Information Table S2.

### Sleep Is Associated with Stronger Functional Connections

**Figure 1.**
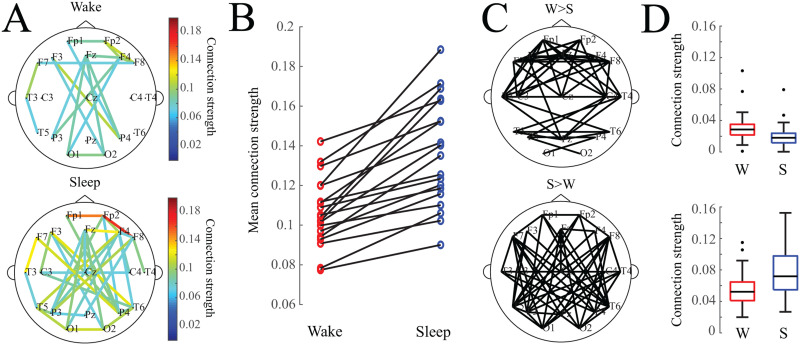
(A) Average functional connectivity networks for wakefulness and sleep. For visualization, an edge is drawn if the connection value exceeds an absolute threshold of 0.075. (B) Mean connection strength for individual subjects (calculated as the average strength of the strongest 10% of connections) is higher during sleep. (C) Network maps showing connections that were statistically significantly greater in wakefulness (top) or sleep (bottom). (D) Box plots of mean connection strength for connections that were significantly different between wakefulness and sleep (shown in subfigure C).

Sleep was associated with stronger functional connections, as evidenced by the averaged connectivity networks ***Q***_*w*_ and ***Q***_*s*_ across all subjects (Figure 1A) and the individual subject results (Figure 1B). We also tested whether specific connection pairs were consistently stronger in wakefulness or sleep; to do this, we compared the distribution of connection strengths for one electrode pair during wakefulness to the distribution during sleep in a pairwise fashion (*n* = 19 subjects). In 48 of the 171 possible connections, wakefulness revealed stronger connectivity values (Figure 1C, top) (two-tailed Wilcoxon sign-rank test, adjusted via Benjamini–Hochberg procedure, adj. *p* < 0.05). In 55 of the 171 possible connections, sleep connectivity values were significantly stronger than wakefulness (Figure 1C, bottom) (two-tailed Wilcoxon sign-rank test, adjusted via Benjamini–Hochberg procedure, adj. *p* < 0.05). The strongest connections in the averaged network were typically associated with sleep rather than wakefulness; although a number of connections were statistically stronger during wakefulness than sleep, these connections were typically weak, with strengths <0.04 (Figure 1D).

### Functional Network Structures Are Subject Specific

**Figure 2.**
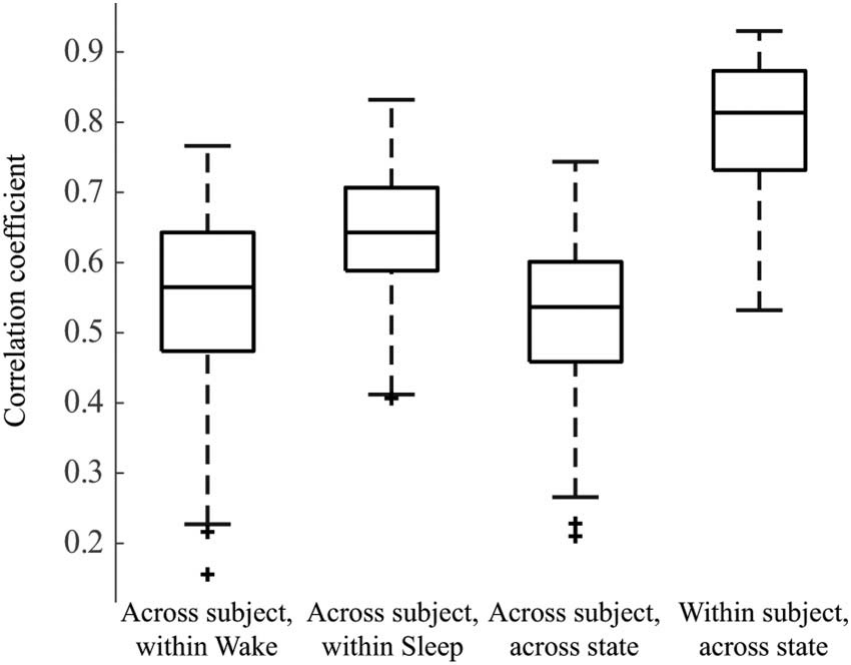
Box plot showing 2-D correlations within and across weighted connectivity matrices for each subject. We first compared networks across subjects within a state, for example, Subject 1 Wake to Subject 2 Wake, and the analogous comparisons during sleep; *n* = 171 observations each. Then we compared across subjects and across states, for example, Subject 1 Wake to Subject 2 Sleep; *n* = 171 observations. Lastly, we calculated the 2-D correlation between the sleep and wake networks within single subjects, for example, Subject 1 Wake to Subject 1 Sleep; *n* = 19 observations. All distributions are statistically significantly different from one another (Wilcoxon rank-sum test, *p* < 0.05).

We then measured the similarity of the functional connectivity network structure within and across subjects, as well as within and across sleep/wake states (Figure 2). Across all subjects, the within-sleep distribution of correlation coefficients was statistically significantly higher than the within-wakefulness distribution, and both were significantly higher than the across-state distribution (Wilcoxon rank-sum test, *p* < 0.05). However, Cohen’s effect size value between the within-wakefulness and across-state distributions suggested low practical significance (*d* = 0.233). All effect sizes are reported in Supporting Information Table S3. Within-subject across-state correlations (e.g., comparing subject 1 sleep to subject 1 wakefulness) were higher than across-subject within-state correlations (e.g., comparing sleep networks across all subjects). This indicates that, while state-specific functional network structure commonalities are seen across subjects, the network structures are also patient-specific.

### Network Structure Is More Segregated During Wakefulness

**Figure 3.**
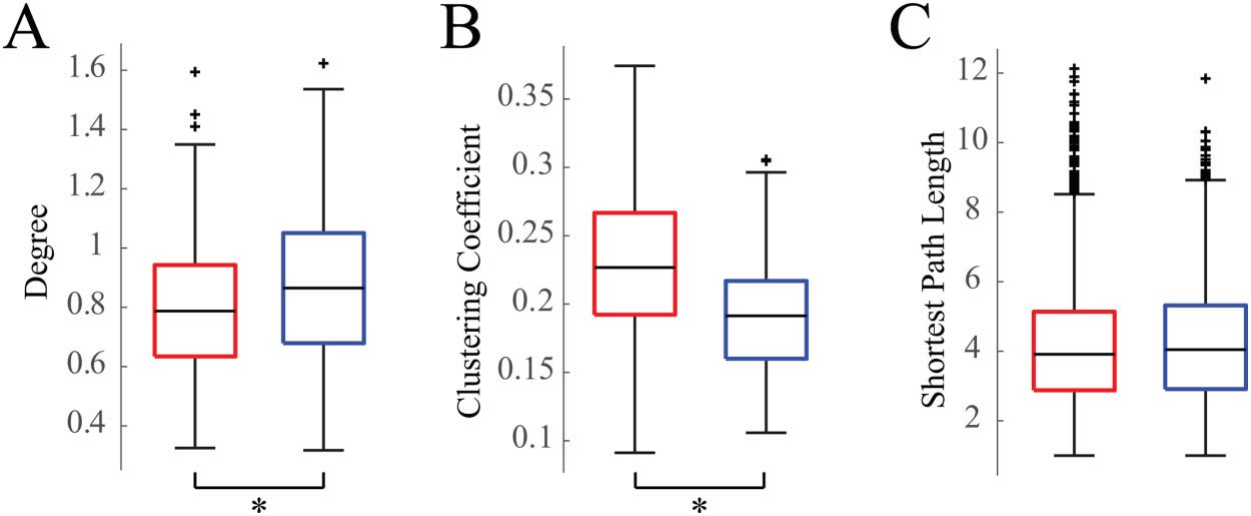
Box plots of weighted graph theoretical measures. (A) Degree, (B) clustering coefficients, and (C) shortest path lengths of wakefulness (red) and sleep (blue) networks for all 19 subjects. Asterisk denotes *p* values less than 0.05.

We calculated three standard weighted graph theoretical measures on the average functional connectivity maps for sleep and wakefulness for each subject. Consistent with our finding that networks tended to be stronger during sleep than during wakefulness, the node degree was significantly greater in sleep than in wakefulness (Figure 3A) (Wilcoxon rank-sum test, *p* < 0.05, Cohen’s *d* = 0.34). The clustering coefficient was significantly greater in wakefulness compared to sleep (Figure 3B) (Wilcoxon rank-sum test, *p* < 0.05, Cohen’s *d* = 0.78). Lastly, we found that the shortest average path length was not significantly different between sleep and wakefulness (Figure 3C) (Wilcoxon rank-sum test, *p* < 0.05, Cohen’s *d* = 0.03).

### Functional Networks Are More Stable in Sleep Than Wakefulness

**Figure 4.**
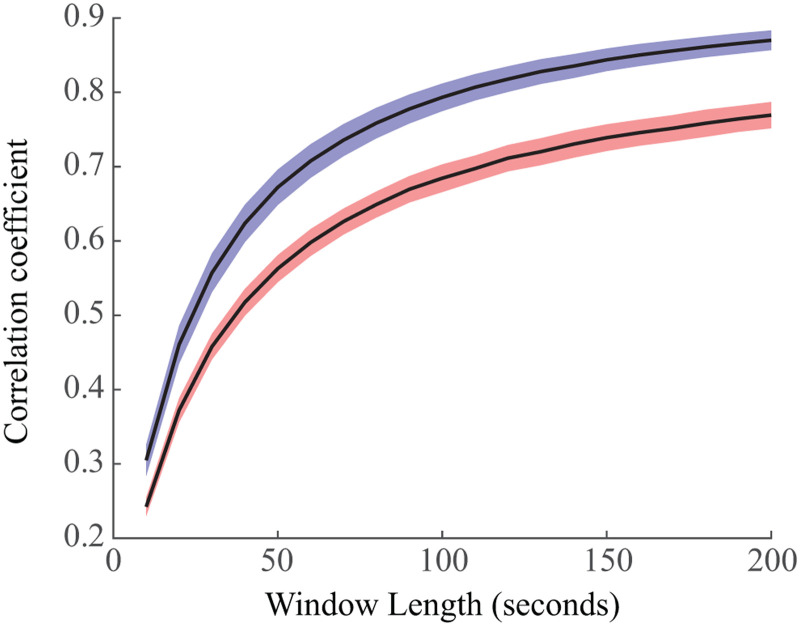
Stability of functional connectivity networks in wakefulness (red) and sleep (blue). We calculated the 2-D correlation between independent averaged connectivity networks from windows of data of varying size. We found that sleep exhibited more stable networks, with nonoverlapping confidence intervals for the means for all window sizes.

We then calculated the stability of each subject’s functional connectivity networks within a given brain state (Figure 4). Here, stability was assessed using the correlation coefficient between networks calculated from independent windows of data. Higher correlation coefficients indicated greater similarity between networks and thus, higher stability. For each subject, we calculated the mean correlation coefficient for each window size and then calculated the 95% confidence interval for the mean correlation coefficients across all subjects. We found that sleep networks were significantly more stable than networks derived from EEG during wakefulness (Figure 4). The confidence intervals for the mean of the stability distributions did not overlap for any window size, indicating statistical significance over all tested window sizes.

### Functional Networks Associated with Sleep and Wakefulness Recur over Hours and Days

Our analysis thus far has shown that an individual subject’s functional network remains stable throughout each period of wakefulness or sleep. However, this analysis did not test whether the networks recur, that is, whether the functional network of one sleep period matches that of another sleep period within the same person. To address this, we used PCA to determine the latent variable that described the most variance in the connectivity data; we hypothesized that this variance would be due to transitions between wakefulness and sleep. If the networks remain stable for each state over multiple sleep/wake cycles, the time series of the first principal component, which signifies the weight of that component in the functional connectivity time series, should oscillate between two values.

**Figure 5.**
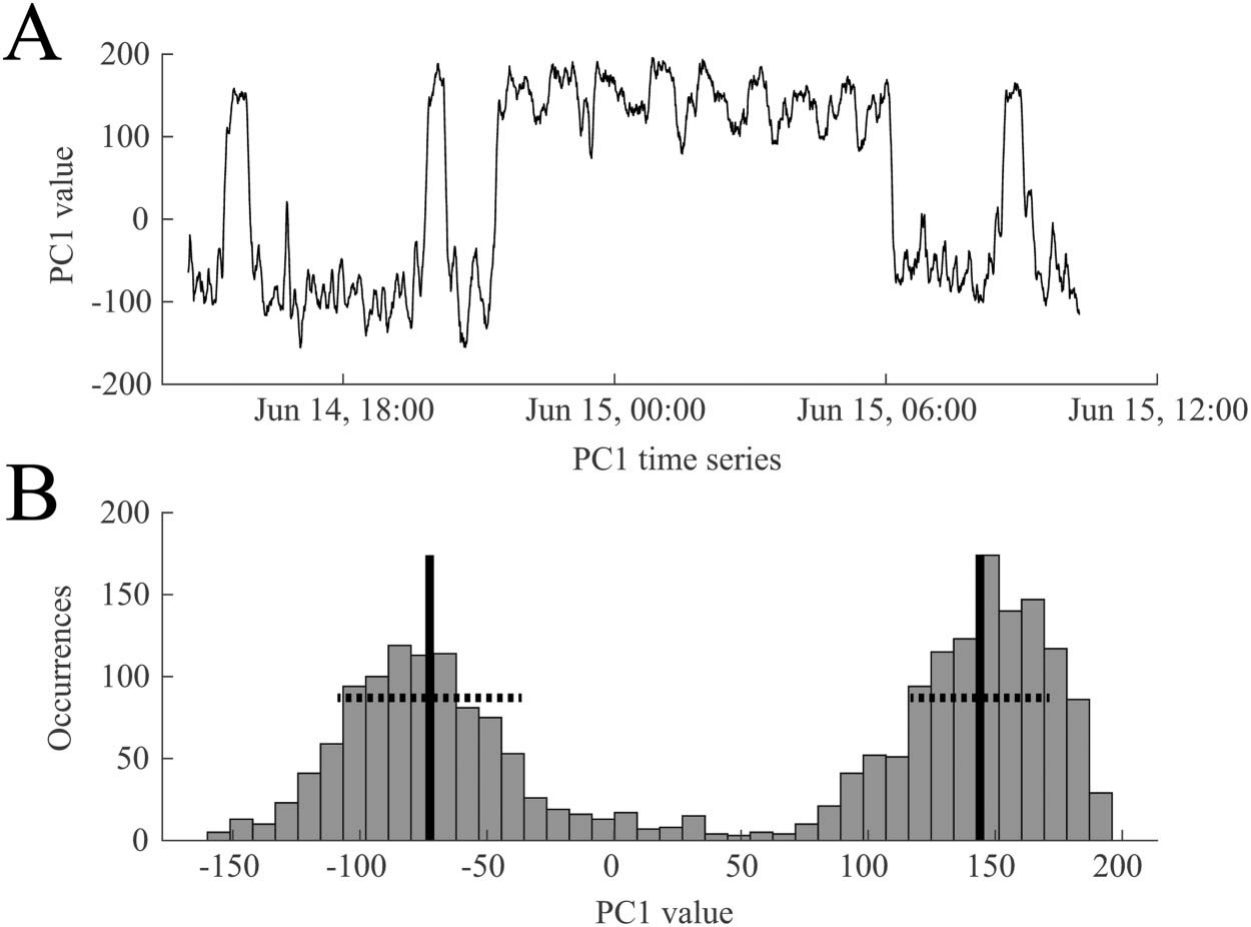
A representative example of the time course of the first principal component (PC1), reflecting how much weight is assigned to PC1 in the functional connectivity time series. (A) PC1 oscillates between two states during ∼18 hr of EEG data. (B) The bimodal nature of PC1 is reflected in its histogram. A two-component Gaussian mixture model was derived from these values and used to classify the two states. The black vertical lines indicate the means of the two distributions, and the dashed horizontal lines denote one standard deviation. Data are from Subject 1.

We performed PCA on all functional connectivity network time series as outlined in section Recurrence of Network States and Correspondence to Manual Sleep Staging. A representative example of a time series from the first principal component (PC1) is shown in Figure 5A, demonstrating the bimodal nature of the signal. This suggests that the brain is switching between two functional network states over the course of 1 day. This was also reflected in the histogram of the first principal component, which we fit with a two-component GMM (Figure 5B).

**Figure 6.**
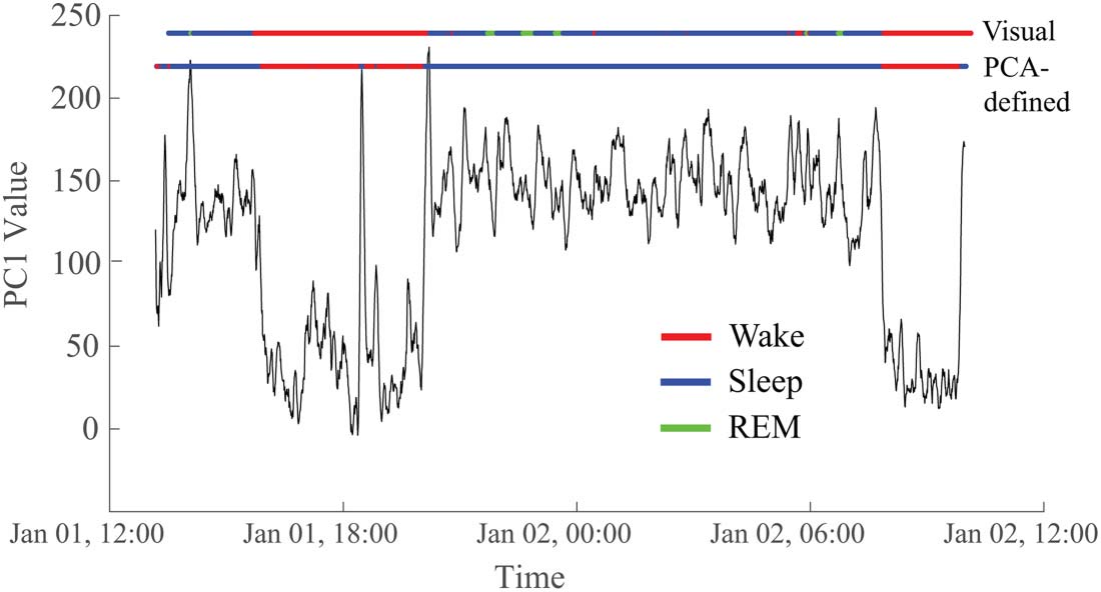
The two states derived from the PC1 time series correspond to visually marked sleep and wakefulness in the EEG. In this representative example, the correspondence is 95.1%. The top horizontal line (“Visual”) is colored to indicate the sleep/wake state based on visual markings. Red indicates the subject is awake, blue is non-REM sleep, and green is REM sleep. The bottom horizontal line (“PCA-defined”) reflects the values of the first principal component after thresholding based on the Gaussian mixture model, with red representing wakefulness and blue representing sleep. Data are from Subject 5.

To determine whether the two reoccurring states evident in the PCA results corresponded to wakefulness and sleep, we compared the GMM output to visual sleep staging (Figure 6). We found a high correspondence between the two states uncovered via PCA (defined by a threshold applied to the GMM) and the visually identified sleep and wake stages. Across subjects, the median percentage of correspondence was 91.2% (Supporting Information Table S4), confirming our hypothesis that the PCA-determined states reflected wakefulness and sleep.

### EEG Functional Networks Exhibit Circadian Variation

**Figure 7.**
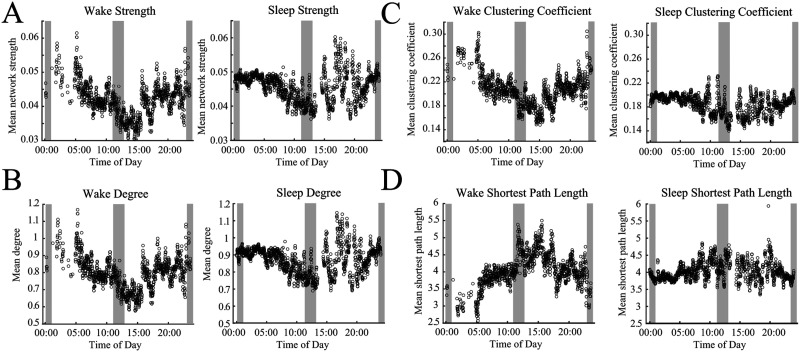
Circadian patterns emerge in both wakefulness and sleep for network strength and graph theoretical metrics. Twenty-four hour periodicities are shown for (A) the network strength, defined as the mean of all connections, (B) network degree, (C) clustering coefficient, and (D) the shortest path length. Each subfigure shows data recorded during wakefulness (left) and sleep (right). Gray shaded regions mark daytime (11:00–13:00) and nighttime (23:00–01:00) hours.

When subjects were awake, the mean network strength was significantly decreased during the daytime and was increased at nighttime (Figure 7A; 95% confidence interval does not include 0). Similar patterns were seen for the network degree (Figure 7B) and the clustering coefficient (Figure 7C). The shortest path length showed the opposite trend (Figure 7D). Similar patterns were observed using data collected when subjects were sleeping, although the modulation over 24 hr was less dramatic. However, all trends in graph theoretical measures were significantly different between daytime and nighttime hours (Figure 7B–7D; 95% confidence interval does not include 0). This indicated that, in addition to the significant differences between sleep and wake functional networks, the time of day modulated the network within each state.

We noted that the sleep measurements during the daytime exhibited high amounts of variability, and, in general, there was higher variance in the measurements when subjects were asleep during the daytime or awake at nighttime. However, this may be partially due to the low number of data points in some time periods, as the high variance did not occur when there were a large number of data points in the 1-min windows (Supporting Information Figure S1). A larger cohort or more EEG data per subject would be needed to robustly quantify this variance.

## DISCUSSION

In this study, we report characteristics of functional connectivity networks based on long-term EEG recordings from 19 healthy infants. We first showed that functional connectivity networks associated with sleep and wakefulness exhibited statistically significant differences both in strength and structure. We also showed that, although sleep and wakefulness were associated with stable networks, those networks were highly individualized. Within-subject comparisons of sleep to wakefulness were more similar than comparisons across subjects within the same state of consciousness. Furthermore, state-specific networks recurred over multiple periods of sleep and wakefulness within each subject, as revealed by the correspondence of PCA-derived networks to visual sleep scoring. Lastly, we showed that circadian rhythms significantly modulated network properties in a relatively stereotyped fashion. This suggests that the time of day during which a recording is obtained may significantly impact measurements of functional connectivity, which bears relevance for both cognitive and clinical studies involving functional networks.

The analysis of infant EEG, which is visually distinct from adult EEG, is a unique aspect of this study. The vast majority of EEG functional connectivity studies focus on healthy adult data (Stevens, 2009); thus, this study fills a critical need by reporting basic characteristics of healthy EEG-based functional networks as a baseline for studying conditions specific to the neonatal/infant period, including early-onset epilepsies and neurodevelopmental conditions (Righi et al., 2014; Shrey et al., 2018). Infant EEG poses unique advantages and disadvantages in comparison with adult recordings. On one hand, it enables study of circadian dynamics and network characteristics separately for sleep and wakefulness because infant sleep cycles do not always coincide with diurnal rhythms. On the other hand, the EEG patterns associated with sleep and wakefulness in infant EEG rapidly evolve as the infant grows and develops. Wakeful background activity in infant EEG is slower than adult EEG, and rhythms become faster with age (Fisch, 1999; Rowan & Tolunsky, 2003). The emergence of critical patterns, such as the posterior dominant rhythm and the mu rhythm, occurs at around 3 and 4–6 months of age, respectively (Laoprasert, 2011; Stern, 2005). Moreover, the structural connectivity in the infant brain is constantly changing and developing (Barkovich et al., 2006; Tymofiyeva et al., 2012), whereas structural connectivity in adults is relatively static. This age dependence could partially explain the subject-specific nature of the networks observed in our study, but we found no significant correlations in our strength and topology metrics (Supporting Information Figure S2), and subject-specific networks were also reported in adults (Chu et al., 2012). While researchers have advanced the study of the relationship between structure and function in the brain (Pernice et al., 2011; Ponten et al., 2010), further work is needed to examine this relationship in the developing brain. Overall, we expect that the functional connections underlying infant neural activity will differ from adults.

We found that functional networks were stronger during sleep than wakefulness, and they were less clustered when the subject was awake (Burroughs et al., 2014; Kuhnert et al., 2010; Mitsis et al., 2020). The networks were significantly stronger during sleep when compared to wakefulness in all subjects (Figure 1B); however, we note that the effects of thresholding networks, even with a proportional threshold, is an active area of research and will require further investigation (Chapeton et al., 2017; Garrison et al., 2015). To reduce the bias introduced by thresholding the network graphs, we calculated graph theoretic properties of the networks on the weighted adjacency matrices. The degree was significantly higher in the functional networks derived from sleep, consistent with our finding of overall stronger networks in sleep. The clustering coefficient of the normalized networks was higher in awake networks, indicating greater functional segregation during wakefulness (Rubinov & Sporns, 2010; Watts & Strogatz, 1998). Interestingly, the shortest path length, calculated as the inverse of the normalized connection strength, was not significantly different between the two states, indicating similar levels of functional integration in wakefulness and sleep (Watts & Strogatz, 1998).

We found that the awake and sleep networks were more similar within a single subject than the awake or asleep networks across subjects. The subject-specific nature of these functional networks was also described in a long-term intracranial EEG study in adults (Kramer et al., 2011), as well as in a previous study by our group in a cohort of pediatric epilepsy patients (Shrey et al., 2018). This may indicate a need for a paradigm shift in the analysis of functional connectivity networks. Most functional network studies have focused on finding common networks and pathways that facilitate specific functions or the resting state. However, the uniqueness of functional networks has become a recent area of investigation in the fMRI community and may deserve further attention in EEG functional network analysis (Chapeton et al., 2017; Chen et al., 2008; D’Esposito, 2019; Dubois & Adolphs, 2016; Fingelkurts & Fingelkurts, 2011; Finn et al., 2020; Gonzalez-Castillo et al., 2015; Kramer et al., 2011). A comparison of functional connectivity networks may require attention to both elements: the common pathways underlying the activity of interest, as well as the individuality of the subject’s unique functional network.

The stability of functional connectivity networks in EEG is a function of the timescale used to measure them. We found that the binary connectivity matrices in 1-s epochs were highly variable, but stable networks were identified over the course of 200–500 s (Chu et al., 2012; Shrey et al., 2018). However, these networks become unstable again at the timescale of hours due to brain state transitions and circadian rhythms. This multilevel stability is assumed in our study, but further investigation is needed to define characteristic timescales of stability in functional connectivity networks in the human brain (Kuhnert et al., 2010). This is perhaps related to the concept that EEG amplitude modulations do not have a characteristic scale and exhibit a fractal nature (Hardstone et al., 2012; Linkenkaer-Hansen et al., 2001; Smith et al., 2017). This fractal nature may be transferred to functional networks (Lehnertz et al., 2017), mathematically suggesting that brain activity is changing in an organized way that may not have a characteristic timescale.

Several limitations of our study should be addressed in future investigations of healthy functional connectivity networks. First, our EEG recordings were an average of 20.8 hr, and a limited number of recordings were longer than 24 hr. Thus, circadian patterns were assessed on the group level rather than an individual level. Future studies could include longer, multiday EEG recordings to analyze true subject-specific assessments of circadian patterns. Second, we used an automatic algorithm to remove artifacts in our data, as it was infeasible to visually confirm all artifacts due to the long recording durations. Therefore, some artifacts may have escaped detection/removal while other artifact-free data may have been erroneously removed. This could have contributed to the differences seen in the wake and sleep networks because artifacts are more frequent during wakefulness; on the other hand, the results reported here mirror those obtained with visually marked artifacts (Shrey et al., 2018). Lastly, although there are many advantages to analyzing data from a cohort of infants, the limited age range reduces the generalizability to other pediatric populations, and we did not have enough subjects to discern network properties specific to each age or developmental stage. Note, though, that a preliminary analysis revealed no modulation in strength or topology metrics as a function of age (Supporting Information Figure S2). Future studies should increase the number of subjects and broaden the age range.

The importance of this study lies in generating functional connectivity networks derived from ∼24-hr recordings of normal EEG data in infants. In addition to imparting knowledge of how physiological functional networks are modulated throughout the day and within waking and sleep states, this will facilitate understanding of changes in network topology due to pediatric diseases such as epilepsy and autism (Righi et al., 2014; Shrey et al., 2018). Seizure forecasting in epilepsy has largely relied on prediction of seizure onset with several minutes of data, but it has been shown that modulations in functional networks due to physiological processes such as waking and sleeping can mask “pre-seizure” changes (Kuhnert et al., 2010; Mitsis et al., 2020; Schelter et al., 2011). Accounting for these physiological fluctuations in seizure prediction models may improve their accuracy and ultimately improve care for patients suffering from epilepsy.

## ACKNOWLEDGMENTS

The authors thank the EEG technologists at the Children’s Hospital of Orange County (CHOC) for their help in acquiring the EEG data. The authors also thank Dr. Michael Nunez and Derek Hu for helpful discussions regarding the manuscript. This study was funded by a CHOC PSF Tithe grant.

## SUPPORTING INFORMATION

Supporting information for this article is available at https://doi.org/10.1162/netn_a_00194.

## AUTHOR CONTRIBUTIONS

Rachel June Smith: Conceptualization; Data curation; Formal analysis; Investigation; Methodology; Project administration; Software; Writing – original draft. Ehsan Alipourjeddi: Data curation; Formal analysis; Investigation; Software; Writing – review & editing. Cristal Garner: Data curation. Amy L. Maser: Data curation. Daniel W. Shrey: Conceptualization; Data curation; Funding acquisition; Methodology; Supervision; Writing – review & editing. Beth A. Lopour: Conceptualization; Funding acquisition; Investigation; Methodology; Project administration; Resources; Supervision; Writing – review & editing.

## FUNDING INFORMATION

Daniel W. Shrey, Children’s Hospital of Orange County, Award ID: PSF Tithe Grant.

## TECHNICAL TERMS

Infant: A pediatric subject typically between 3 and 12 months of age.
State of consciousness: The subject’s level of awareness; here, we specifically study the states of wakefulness and sleep.
Ultradian periodicities: Fluctuations in network characteristics with periods greater than 1 hr and less than 24 hr, such as the sleep cycle.
Sleep staging: Visual categorization of EEG into wakefulness, rapid eye movement (REM) sleep, and non-REM sleep stages 1–3; also called sleep scoring.
Cross-correlation: Bivariate, amplitude-based synchronization measure, calculated as the dot product of two EEG signals as one signal is shifted in time.
Network strength: Amount of synchronization between nodes, here defined as the percentage of 1-s epochs in which two nodes are significantly connected.
Principal component analysis: Dimensionality reduction technique based on the eigendecomposition of the covariance matrix.
Gaussian mixture model: Parametric model that assumes the data can be represented by a weighted sum of a finite number of Gaussian distributions.
Network stability: Variability of the functional network within a single state of consciousness; stable networks are highly correlated over time.
Topology metrics: Graph theoretical measurements of network characteristics, such as degree, clustering coefficient, and shortest path length.
