# Supplementary material for: Infant functional networks are modulated by state of consciousness and circadian rhythm
Source: Netw Neurosci. 2021 Jun 21;5(2):614–30. doi: 10.1162/netn_a_00194 (PMC8233111; doi:10.1162/netn_a_00194)
Supplement: Supplementary file 1 [file netn-05-614-s001.pdf]

Smith, R. J., Alipourjeddi, E., Garner, C., Maser, A. L., Shrey, D. L. & Lopour, B. A. (2021). Supporting information for "Infant functional networks are modulated by state of consciousness and circadian rhythm". *Network Neuroscience*. Advance publication. https://doi.org/10.1162/netn_a_00194

Supplementary Material

Supplementary Table 1: Percentage of epochs removed due to suspected volume conduction

Subject Number	Percentage of epochs removed for maximum cross-correlation at 0 lag
Subject 1	28.11
Subject 2	28.84
Subject 3	30.26
Subject 4	26.8
Subject 5	31.44
Subject 6	31.15
Subject 7	27.53
Subject 8	28.39
Subject 9	32.41
Subject 10	27.92
Subject 11	30.04
Subject 12	27.73
Subject 13	31.77
Subject 14	29.9
Subject 15	31.02
Subject 16	29.56
Subject 17	32.09
Subject 18	30.05
Subject 19	30.07

Supplementary Table 2: Recording durations and sleep/wake durations per subject

Subject Number	Total recording duration (seconds)	Duration wakefulness (seconds)	Duration sleep (seconds)
Subject 1	60459	19779	27773
Subject 2	67405	31922	34412
Subject 3	65091	24481	39469
Subject 4	50031	12535	37466
Subject 5	71251	23172	43274
Subject 6	57076	11649	31734
Subject 7	149185	64722	74188
Subject 8	75745	22911	47953
Subject 9	79154	36317	41475
Subject 10	90858	37119	43994
Subject 11	128809	47223	68756
Subject 12	57096	16816	39852
Subject 13	58928	17646	40551
Subject 14	49957	15502	34326
Subject 15	50565	14621	24702
Subject 16	120379	56180	60073
Subject 17	56964	23801	30283
Subject 18	69497	17079	51985
Subject 19	63126	19173	43905

Supplementary Table 3: Effect sizes for correlation coefficients between connectivity matrices

Compared distributions	Cohen’s d (effect size)
(Across patient, within wake) vs. (Across patient, within sleep)	0.874
(Across patient, within wake) vs. (Across patient, across state)	0.233
(Across patient, within wake) vs. (Within patient, across state)	2.227
(Across patient, within sleep) vs. (Across patient, across state)	1.244
(Across patient, within sleep) vs. (Within patient, across state)	1.637
(Across patient, across state) vs. (Within patient, across state)	2.724

Supplementary Table 4. Automatic identification of sleep/wake state for 19 subjects

	Scored Wake/ Classified Wake (%)	Scored Sleep/ Classified Sleep (%)	Scored Sleep/ Classified Wake (%)	Scored Wake/ Classified Sleep (%)	Percent Correctly Classified
**Subject 1**	29.38	54.37	5.14	11.09	**83.75**
**Subject 2**	45.90	52.92	0	1.16	**98.83**
**Subject 3**	32.68	62.48	0	4.82	**95.17**
**Subject 4**	24.60	24.54	50.84	0	**49.15**
**Subject 5**	28.21	67.46	0.29	4.02	**95.67**
**Subject 6**	21.93	74.98	0.13	2.94	**96.92**
**Subject 7**	24.44	56.47	0.06	19.02	**80.91**
**Subject 8**	31.47	18.94	49.58	0	**50.41**
**Subject 9**	38.68	53.21	0.79	7.30	**91.89**
**Subject 10**	31.96	55.26	0	12.77	**87.22**
**Subject 11**	34.58	59.58	0.02	5.79	**94.17**
**Subject 12**	25.86	32.66	37.75	3.71	**58.52**
**Subject 13**	28.59	23.57	46.27	1.55	**52.17**
**Subject 14**	30.69	60.54	8.15	0.60	**91.23**
**Subject 15**	27.92	63.73	0	8.33	**91.66**
**Subject 16**	43.82	53.07	0.20	2.90	**96.89**
**Subject 17**	36.57	56.87	0.16	6.38	**93.44**
**Subject 18**	24.20	12.75	62.60	0.43	**36.96**
**Subject 19**	22.72	56.70	13.46	7.11	**79.42**

Subjects in which the percentage of correctly classified epochs exceeded 80% are highlighted in green (n=13 subjects). Subjects in which greater than 15% of the data were incorrectly classified are highlighted in red (n=6 subjects).

Supplementary Figures:

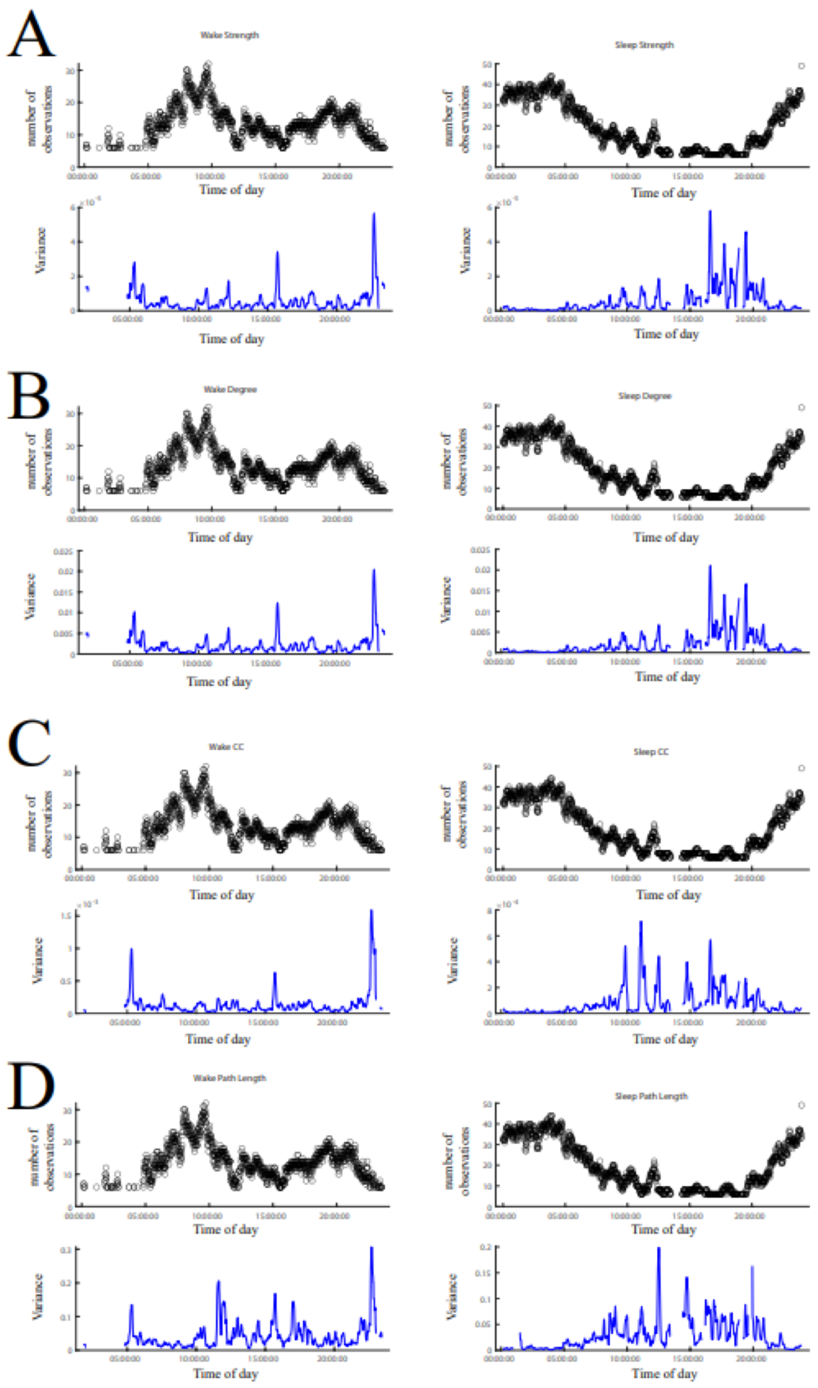

Supplementary Figure 1. The number of observations of strength and topology metrics over ~24-hour periods is related to the variance in the mean values.

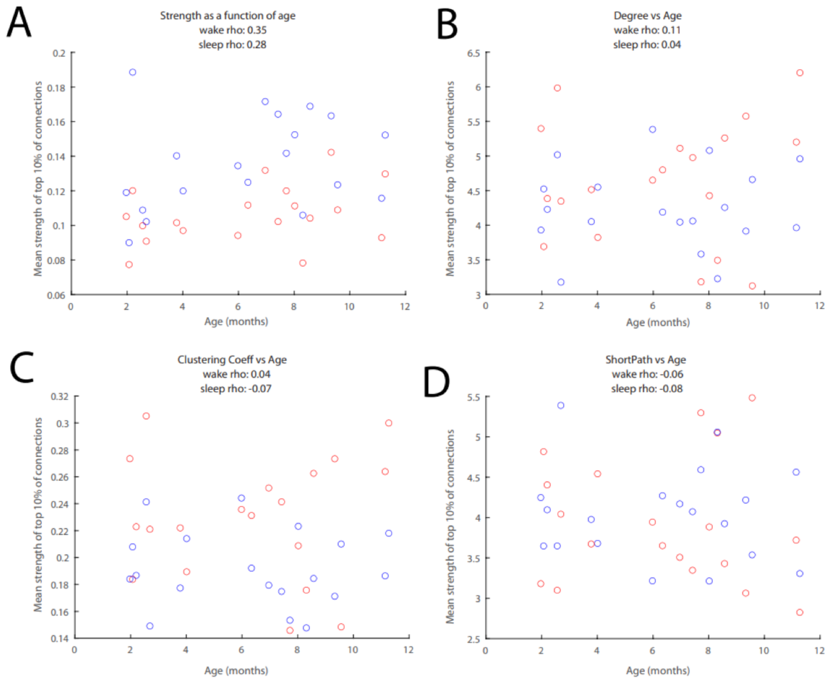

Supplementary Figure 2. Mean functional connectivity strength (mean top 10% of connections) and topology metrics as a function of age (months). Network strength during wakefulness was slightly correlated with age (rho = 0.35). Network strength during sleep was even less correlated with age (rho = 0.28). The topology metrics did not significantly vary with age.
